# Shared decision-making should be a standard part of surgical care

**DOI:** 10.1093/bjs/znac291

**Published:** 2022-09-06

**Authors:** Dirk T Ubbink

**Affiliations:** Department of Surgery, Amsterdam University Medical Centers, location University of Amsterdam, Amsterdam, The Netherlands

Shared decision-making is here to stay as a means to further high-quality care in modern surgery. This is fostered by current societal and professional developments. More and more treatment options (e.g. drugs, and laparoscopic and robotic techniques) become available and (future) patients can more easily and digitally access medical information about their disease and treatment options.

Today’s surgeons, as knowledgeable and skilled experts, offer state-of-the-art care using cutting-edge surgical techniques, apply current evidence-based clinical practice guidelines, discuss complex cases in multidisciplinary team meetings in order to decide on the best possible treatment, and, finally, share this outcome with their patients to achieve informed consent. Their advice regarding a certain treatment option is based on expertise, appreciation of the patient’s condition, and known evidence about the expected desired or unwanted effects.

However, this advice may well be flawed until surgeons acknowledge the patient’s autonomy and explicitly involve the patient’s expectations regarding their goals in life and preferences for a certain treatment—even if these are not the surgeon’s first choice. This requires bidirectional communication between healthcare professionals and patients to elicit and appreciate the patient’s preference, and incorporate this in the eventual treatment choice^1^. This process is called shared decision-making (SDM).

SDM is particularly valuable in surgery^2^: surgical treatments always run the risk of complications, which may cause clinical decision-making to be quite intricate. Elective interventions may cause immediate harm (i.e. peri- and postoperative complications), while the desired effects (e.g. prevention of death due to cancer) may take years to be seen. In elderly patients or those with multimorbidity, surgical interventions may be even less appealing.

Still, many surgeons find SDM difficult, both conceptually and in clinical practice. Most will not have been educated in the principles and practice of SDM. However, SDM is not a completely new paradigm; it is an essential part of the definition of evidence-based medicine (EBM) since the 1990s^3^. Surgeons have become familiar with EBM, but not (yet) with involving patients’ preferences. It is an ethical and legal obligation to inform a patient in detail about the expected desired and possible undesired outcomes of any possible intervention before asking for informed consent. It is equally essential to consider the patient’s preference when deciding on treatment options^4^. These preferences help determine whether the available evidence on the possible benefits and harms of the surgical treatment options match the patient’s situation and preferences. The outcome measure the surgeon finds decisive may be different from the outcome that is most relevant to the patient. The surgeon may advise surgery as it may prolong life, whereas the patient may prefer fewer interventions, hospital stays, and visits, and therefore a better quality of life, even at the cost of a shorter survival time.

Surgical patients also prefer SDM^5^. When given the choice, they tend to prefer a less, or even non-invasive, treatment option^6^. Hence, this may reduce overuse of surgical care. Patients are unlikely to make a ‘wrong’ choice, as long as surgeons discuss the pros and cons of each option (including ‘doing nothing’, or mere surveillance), and patients are invited to share their opinions and preferences regarding these options. In general, patients are in favour of this approach as it is all about their bodies and their lives. Moreover, patients are more satisfied, and have more knowledge and less decisional conflict when they are involved in the decision-making process^7,8^. In turn, surgeons will be happier when sharing the burden of responsibility regarding a treatment choice, achieving a better relationship with their patients, and being less at risk of liability issues.

Surgeons may be inclined to follow clinical practice guidelines and therefore be reluctant to engage in SDM. Guidelines provide valuable evidence-based recommendations, but they do not give any clue as to a patient’s situation, goals, and preferences. Hence, there may be a good reason to deviate from a guideline.

Obviously, not all patients will want to collaborate in the decision-making process. ‘What would you do doctor?’ is an often-heard question that cannot be answered, because the surgeon’s preferences usually do not coincide with the patient’s. Patients should at least be informed that there are options to choose from and be invited to express their ideas or concerns about different treatment options. Some patients cannot express their preferences (e.g. if they have cognitive impairment or are unconscious). Then, the patient’s partner or relatives may share what they would prefer. Another—quite large—group of patients have a low health literacy. Although patients may not fully understand or imagine what the (consequences of) the options are, it is the surgeon’s task to find out what matters to the patient.

To facilitate this process, surgeons will need to adapt their consultation habits to encourage SDM. Some will say they already do it, and fortunately so. However, a large amount of unconscious incompetence exists^9^. They may inform their patients thoroughly and ask them what they want. However, SDM is not about who takes the decision, but the process of arriving at this decision. The most neglected steps in this process are, firstly, telling the patient from the start that a choice has to be made in which their voice is essential. Secondly, after having explained the options, patients should explicitly be asked for their thoughts on these options, as input to a discussion about what option would best match their preference and situation. This may also be discussed together with the patient’s partner or relatives.

Even if there seems to be an obvious single option, the consequences of not choosing this may help the patient to agree with this option. Presenting the information in a neutral fashion for all feasible options is required to avoid framing of the information towards the surgeon’s preference. Also, the available evidence may not be robust. Being honest with patients about this uncertainty makes SDM even more relevant.

Initially, this new approach may take some more consultation time, but eventually it will save time as patients are better informed and have fewer questions later on. Consultation duration may even be reduced when preconsultation decision-making support tools are employed. Many tools are already available to support both surgeons and patients to engage in SDM, for example e-learning, communication skills training, patient decision aids, and option grids^6,10,11^.

Thus, implementing SDM seems to be a win–win situation for all stakeholders involved. Hence, respectful decision-making by surgeons and patients together, while acknowledging they are both experts in their field, should be a standard part of surgical care.
